# Age- and height-adjusted total kidney volume growth rate in autosomal dominant polycystic kidney diseases

**DOI:** 10.1007/s10157-018-1617-8

**Published:** 2018-07-26

**Authors:** Eiji Higashihara, Kouji Yamamoto, Shinya Kaname, Takatsugu Okegawa, Mitsuhiro Tanbo, Tsuyoshi Yamaguchi, Kaori Shigemori, Isao Miyazaki, Kenichi Yokoyama, Kikuo Nutahara

**Affiliations:** 1 Department of Hereditary Kidney Disease Research, Kyorin University Faculty of Medicine, 6-20-2 Shinkawa, Mitaka, Tokyo, 181-8611 Japan; 2 Department of Urology, Kyorin University Faculty of Medicine, Tokyo, Japan; 3 Department of Radiology, Kyorin University Faculty of Medicine, Tokyo, Japan; 4 Department of Nephrology, Kyorin University Faculty of Medicine, Tokyo, Japan; 5 Department of Medical Statistics, Graduate School of Medicine, Osaka City University, Osaka, Japan

**Keywords:** Autosomal dominant polycystic kidney disease (ADPKD), Total kidney volume (TKV), Height-adjusted total kidney volume (HtTKV), Estimated glomerular filtration rate (eGFR)

## Abstract

**Background:**

The Mayo Clinic Image Classification (MIC) was proposed as a renal prognosis prediction model for autosomal dominant polycystic kidney disease (ADPKD). MIC is based on the assumption of exponential constant increase in height-adjusted total kidney volume (HtTKV). HtTKV growth rate is calculated by one-time measurement of HtTKV and age. We named it as an age-adjusted HtTKV growth rate (AHTKV-α). AHTKV-α was compared with HtTKV slope measured by at least two HtTKV values.

**Methods:**

Comparison of repeatability between AHTKV-α and HtTKV slope, correlation of subgroups divided according to baseline AHTKV-α and HtTKV slope with disease manifestations, estimated glomerular filtration rate (eGFR) slope, and renal survival were analyzed in 296 patients with ADPKD. *PKD* genotype influences were compared between AHTKV-α and HtTKV slope in 88 patients with characterized *PKD* mutations.

**Results:**

Absolute differences between baseline and follow-up measures were significantly larger for the HtTKV slope than for AHTKV-α (*P* < 0.0001). From baseline AHTKV-α-based subgroups A–E according to MIC, disease manifestations occurred earlier and future eGFR slopes became steeper (*P* < 0.0001). Multivariate hazard ratios of renal survival differed significantly among baseline AHTKV-α-based subgroups. Inter-subgroup differences in these predictors were less evident during baseline HtTKV slope-based classification. AHTKV-α values, but not HtTKV slopes, were significantly higher for *PKD1* mutation carriers than for *PKD2* mutation carriers (*P* < 0.0001).

**Conclusion:**

MIC is a good renal prediction model applicable to Japanese patients also. AHTKV-α can be a more sensitive and reliable indicator in TKV growth rate than HtTKV slope.

**Electronic supplementary material:**

The online version of this article (10.1007/s10157-018-1617-8) contains supplementary material, which is available to authorized users.

## Introduction

Autosomal dominant polycystic kidney disease (ADPKD) is a hereditary kidney disease that progresses to end-stage renal disease (ESRD) in approximately 50% of patients [1]. Continuous enlargement of total kidney volume (TKV) is a hallmark of ADPKD [1–7]. The Consortium of Radiologic Imaging Studies of Polycystic Kidney Disease (CRISP) indicated that TKV increases in an exponential-like pattern, at different rates among patients but at a constant growth rate within the patient, and that TKV growth can be used as a prognostic marker [4]. Kidney enlargement was associated with a decline in renal function, and TKV slope has been used as an outcome measure in ADPKD clinical trials [8–11]. Eight years follow-up of the CRISP study qualified baseline height-adjusted TKV (HtTKV) as a prognostic biomarker for renal disease progression [6].

Mayo Clinic investigators proposed the Mayo Imaging Classification (MIC) of ADPKD as a renal prognosis prediction model to select patients with rapidly progressive disease for enrollment in clinical trials [12]. When MIC was used, estimated glomerular filtration rate (eGFR) slopes were significantly different from subclasses 1A–1E. The classifications of the MIC model depend on the assumption that HtTKV increases continuously and exponentially at an annual kidney growth rate of *α* (%/year) from HtTKV of 150 mL/m at age 0. MIC subgroups were divided according to the estimated kidney growth rate which is obtained by one-time measurement of HtTKV and age at measurement. We named it as an age-adjusted HtTKV growth rate (AHTKV-α). AHTKV-α was compared with HtTKV slope, which was measured by at least two separated HtTKV values.

In the present study, we validated AHTKV-α by comparison with HtTKV slopes. First, the repeatability of AHTKV-α values and HtTKV slopes were compared. Then, correlations with relevant clinical manifestations, future eGFR slopes, and renal survival were compared between the classifications based on baseline AHTKV-α and baseline HtTKV slope. Influences of *PKD* mutations on the HtTKV growth rate were compared between AHTKV-α and HtTKV slope. The results confirmed that MIC is a useful renal prognosis prediction model and suggested that AHTKV-α can be used as a sensitive and reliable HtTKV growth rate.

## Materials and methods

### Study participants and corresponding study designs

**Fig. 1:**
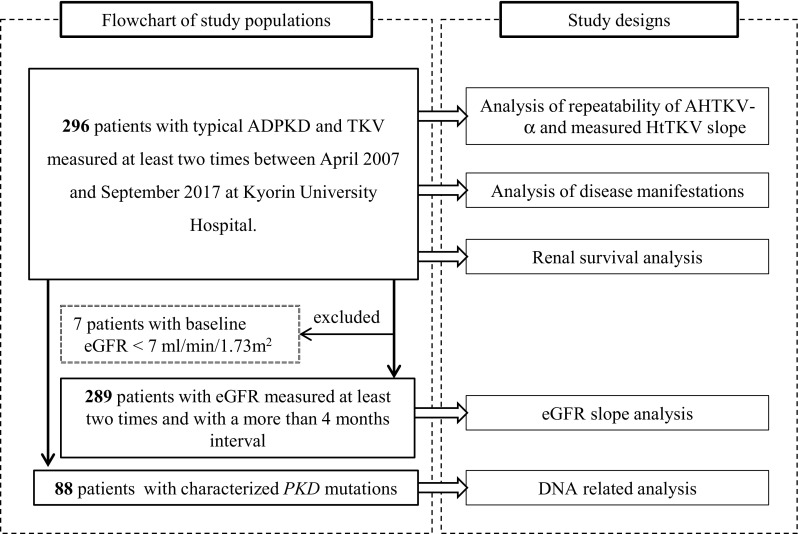
Study populations (in the left dotted box) and corresponding study designs (in the right dotted box) are connected by arrows. Data obtained after tolvaptan administration or surgical renal intervention were excluded from the study

This study was carried out on 296 patients with typical ADPKD and TKV measured two times or more (Fig. 1). Since April 2007, TKV has been measured regularly once per year using the same volumetric method in Kyorin University Hospital [7]. AHTKV-α values were compared with measured HtTKV slope values regarding repeatability. Correlations of disease manifestations and renal survival with MIC, classifications equally divided into five subgroups (A, B, C, D, E) based on baseline AHTKV-α and baseline-measured HtTKV slopes values were compared.

Correlations of eGFR slopes with MIC and other classifications were evaluated for 289 patients. During the eGFR slope analysis, data from patients with baseline eGFR < 7 mL/min/1.73 m^2^ and eGFRs measured after renal replacement therapy (RRT) were not considered.

The influences of *PKD* mutations types on AHTKV-α and measured HtTKV slopes were compared for 88 patients with previously characterized *PKD* mutations [13].

Data obtained after tolvaptan administration or renal surgical intervention was not considered.

### AHTKV-α calculations and measurements of HtTKV slopes

As HtTKV at age *t* (HtTKV_*t*_) is expressed as HtTKV_*t*_ = 150 × (1 + *α*)^*t*^, *α* (= AHTKV-α) is calculated as follows:

$${\text{AHTKV-}}{\upalpha} = ((10^{{({\text{LOG}}10({\text{HtTKV}}_{t} {\text{/}}150))/t)}} ) - 1) \times 100.$$

Measured HtTKV slopes (%/year) were calculated as follows:

$$\begin{aligned} {\text{Measured HtTKV slopes}} & =[{\text{Difference in two consecutive HtTKV}}]/[{\text{HtTKV at an earlier point}}] \\ & \quad /[{\text{Interval (year) of two observations}}] \times 100. \\ \end{aligned}$$

Patients were divided into five subgroups according to MIC using AHTKV-α (%/year): < 1.5 (1A), 1.5–3.0 (1B), 3.0–4.5 (1C), 4.5–6.0 (1D), and > 6.0 (1E) [12]. For comparison purposes, patients were equally divided into five (A–E) or three subgroups using baseline AHTKV-α and baseline-measured HtTKV slope.

### Statistical analyses

Because sex had no significant effect on the analyses of future eGFR slopes in the MIC study [12], the eGFR slopes were evaluated longitudinally using a mixed-effects model for repeated measures depending on age, sex, and interaction between group and age. The following covariance structures were considered: unstructured, compound symmetric, and first-order autoregressive. The covariance structure that provided the best fit according to Akaike’s information criterion was used during the final analysis. To determine the differences between adjacent groups regarding the slopes, Benjamini and Hochberg’s multiple comparison procedure was used and false discovery rate (FDR)-adjusted *P* values were reported [14]. Renal survival rates were analyzed based on three subgroups divided according to the MIC, baseline AHTKV-α and baseline-measured HtTKV slope using the Cox multivariable proportional hazard model for group and sex. Repeatability of HtTKV slopes was examined by the differences in repeated measures according to baseline values and Bland–Altman plots.

Parametric variables are expressed as mean (± SD or ± SE). The effects of subgroups according to AHTKV-α and HtTKV slope on continuous and categorical variables were examined using an analysis of variance, and Pearson’s Chi-squared test, respectively. Hazard ratios (HR) are shown with 95% confidence intervals (CI). All statistical analyses were performed using JMP version 10.0.0 Basic Analysis and Graphing (SAS Institute Inc., Cary, NC) and R version 3.4.1 (R Foundation for Statistical Computing, Vienna, Austria). All tests were two-sided, and *P* < 0.05 and FDR-adjusted *P* < 0.05 were considered statistically significant.

## Results

### TKV and eGFR measurements

TKV and eGFR were measured 1240 and 1495 times, respectively. A summary of the measurements is shown in Supplemental Table 1.

### Comparisons of repeatability between AHTKV-α and measured HtTKV slopes

**Fig. 2:**
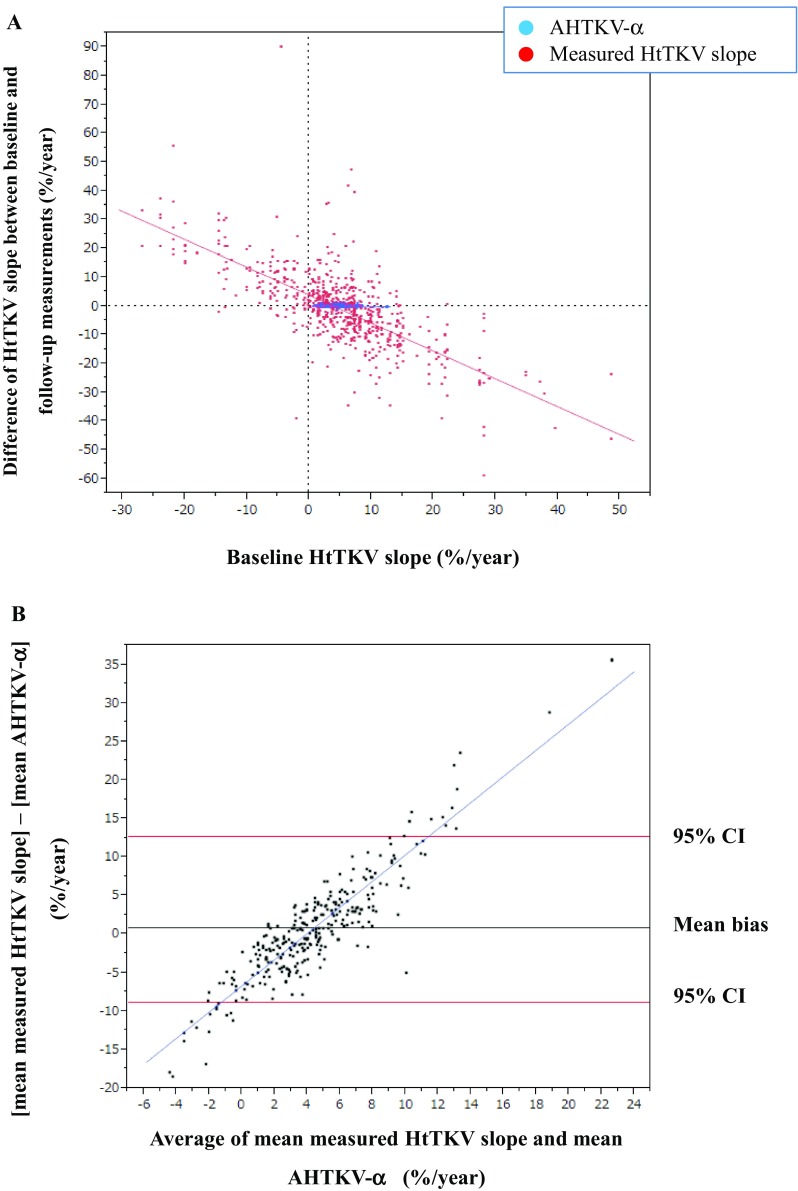
**a** Differences between the baseline and mean follow-up measurements were compared between the AHTKV-α and measured HtTKV slope. The means ± SD of the absolute differences between the baseline and mean follow-up measurements were 0.22 ± 0.21% per year and 9.77 ± 9.48% per year for the AHTKV-α and measured HtTKV slope, respectively (*P* < 0.0001). **b** Bland–Altman plots of the AHTKV-α and measured HtTKV slope. The slope of the plots was significant (*R*^2^ = 0.833, *P* < 0.0001) and indicated a proportionally increased bias between the two measurements

Differences between baseline and follow-up measurements of AHTKV-α and measured HtTKV slopes were plotted against baseline measurements (Fig. 2a). The absolute differences between baseline and follow-up measurements were significantly larger for the measured HtTKV slope than for AHTKV-α (9.77 ± 9.48 versus 0.22 ± 0.21%/year; *P* < 0.0001). Regression slopes for measurement differences between baseline and follow-up measurements were − 0.969 ± 0.952 (*R*^2^ = 0.510, *P* < 0.001) and − 0.016 ± 0.220 (*R*^2^ = 0.0056, *P* = 0.058) for the measured HtTKV slope and AHTKV-α, respectively (difference in slopes, *P* < 0.0001) (Fig. 2a). For the measured HtTKV slope, differences between baseline and follow-up measurements became increasingly negative when baseline values were larger; however, for AHTKV-α, the differences remained within a narrow range (Fig. 2a). The regression slope of the log-converted TKV slope was similar to that of the measured HtTKV slope (Supplemental Fig. 1). Bland–Altman plots showed proportionally increased bias (*R*^2^ = 0.833 and *P* < 0.0001 in Fig. 2b), which was interpreted as increased fluctuation in the measured HtTKV slope. The results suggested good repeatability for AHTKV-α and increased variations corresponding to prior measurement values for repeated measurements of the HtTKV slope.

### Characteristics of MIC and classifications using baseline AHTKV-α and baseline-measured HtTKV slope

**Fig. 3:**
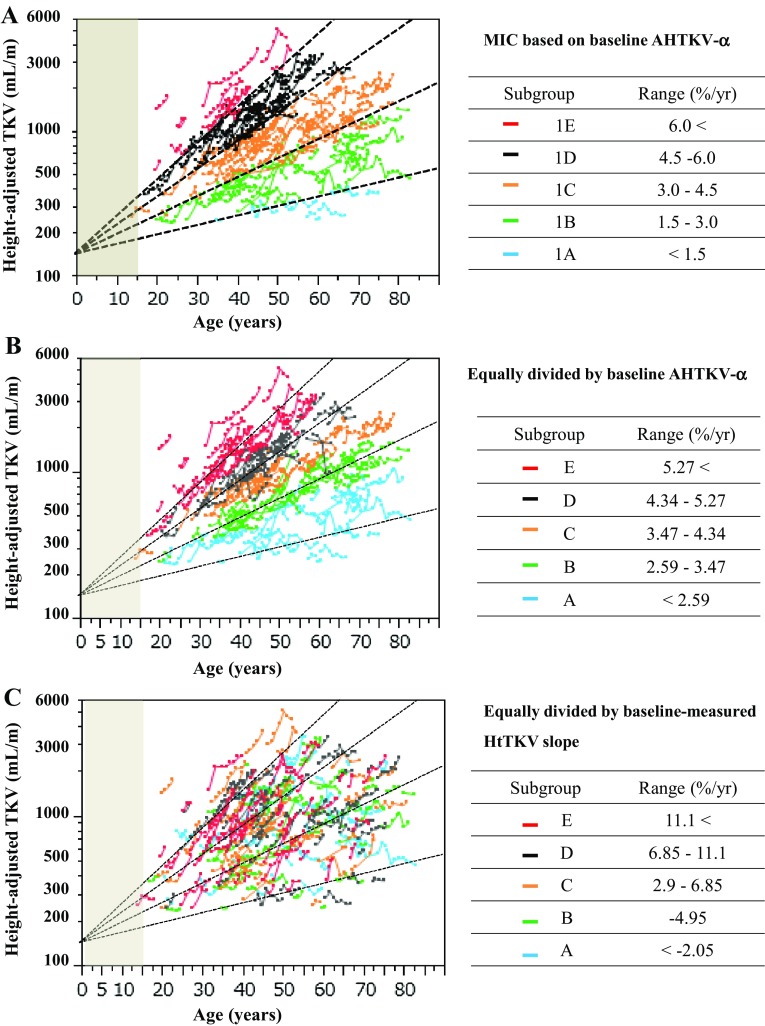
Log-converted HtTKV plotted against age for 296 patients. Four limits (1.5% per year, 3.0% per year, 4.5% per year and 6.0% per year) separated five MIC subgroups. Within-patient plots were connected. The shaded area indicates age younger than 15 years. **a** Five subgroups 1A–1E were classified according to the baseline AHTKV-α and MIC definitions [12]. During the follow-up period, connected plots mostly remained within the same subclass. Patients were equally divided into five subgroups according to baseline AHTKV-α (**b**) and baseline-measured HtTKV slope (**c**). MIC limits (dotted lines) are illustrated for comparison purposes

Scatterplots of the log-converted HtTKV against the ages of the 296 patients are shown in Fig. 3. Five MIC subgroups (1A–1E) were classified using baseline AHTKV-α. Log-converted HtTKV plots against age remained mostly within the same baseline subclass over the course of the years despite considerable inter-patient variations (Fig. 3a). For comparison purposes, similar scatterplots of the five equally divided subgroups using baseline AHTKV-α and baseline-measured HtTKV slope are shown in Fig. 3b, c, respectively. For classification using baseline AHTKV-α (Fig. 3b), within-patient plots were most clearly separated by subgroups; however, for classifications using baseline-measured HtTKV slope, plots were mixed or moved across the limits of the MIC (Fig. 3c).

### Correlations of biomarkers with relevant clinical features

The demographic characteristics of the 296 patients are shown according to MIC and classifications equally divided into five subgroups based on two biomarkers (Table 1). From subgroups A–E, based on MIC and AHTKV-α-based classifications, age at diagnosis, age at onset of disease manifestation and hypertension became significantly younger (all *P* < 0.0001), and the percentage of hypertensive patients increased significantly. In addition, baseline age (corresponding to age at the initial presentation to Kyorin University Hospital) was younger and baseline HtTKV (corresponding to HtTKV during the first measurement) was larger. From subgroups A–E, regarding baseline AHTKV-α-based classifications, disease manifestations became severe and patients visited the hospital earlier. In contrast, the significance of inter-subgroup differences in disease severity was less evident for classification based on the baseline-measured HtTKV slope.

**Table 1:** Demographic characteristics according to MIC and classifications equally divided by two baseline biomarkers

	A	B	C	D	E	*P* value
Range of biomarkers						
MIC based on AHTKV-α (%/year)	< 1.5	1.5–3.0	3.0–4.5	4.5–6.0	≥ 6.0	
Baseline AHTKV-α (%/year)	< 2.59	2.59–3.47	3.47–4.34	4.34–5.27	≥ 5.27	
Baseline-measured HtTKV slope (%/year)	< − 2.05	− 2.05–2.9	2.9–6.85	6.85–11.1	≥ 11.1	
Patient number (male/female)						
MIC based on AHTKV-α	11 (3/8)	69 (19/50)	112 (42/70)	80 (36/44)	24 (13/11)	0.0893
Baseline AHTKV-α	59 (16/43)	59 (15/44)	60 (25/35)	59 (27/32)	59 (30/29)	0.0107
Baseline-measured HtTKV slope	59 (14/45)	59 (22/37)	60 (27/33)	59 (22/37)	59 (28/31)	0.0721
Baseline age (year)						
MIC based on AHTKV-α	55.9 (10.5)	49.6 (14.8)	49.7 (13.3)	40.5 (10.3)	33.6 (7.7)	< 0.0001
Baseline AHTKV-α	51.4 (14.3)	50.8 (14.1)	49.0 (13.4)	43.7 (9.4)	35.8 (10.0)	< 0.0001
Baseline-measured HtTKV slope	49.3 (13.1)	48.4 (14.4)	43.8 (12.9)	47.7 (13.5)	41.5 (13.1)	0.0061
Baseline TKV (mL)						
MIC based on AHTKV-α	485.2 (237.2)	814.0 (94.7)	1634.4 (74.3)	2150.2 (88.0)	2518.3 (160.6)	< 0.0001
Baseline AHTKV-α	694.6 (251.5)	1221.8 (506.3)	1745.7 (800.9)	2053.0 (884.8)	2340.2 (1150.1)	< 0.0001
Baseline-measured HtTKV slope	1672.2 (943.5)	1628.1 (1023.8)	1540.9 (997.7)	1760.2 (1011.0)	1457.3 (903.0)	0.4954
Baseline HtTKV (mL/m)						
MIC based on AHTKV-α	308.1 (52.0)	512.1 (225.3)	1004.8 (459.5)	1296.5 (652.8)	1503.2 (629.4)	< 0.0001
Baseline AHTKV-α	435.6 (163.7)	765.8 (331.8)	1066.4 (498.9)	1243.6 (541.7)	1403.7 (712.5)	< 0.0001
Baseline-measured HtTKV slope	1040.2 (591.5)	1000.2 (618.2)	944.6 (628.5)	1062.5 (602.2)	869.7 (528.3)	0.4023
Age at ADPKD diagnosis (year)						
MIC based on AHTKV-α	53.5 (10.8)	42.7 (12.7)	41.0 (12.6)	32.5 (8.9)	24.0 (7.9)	< 0.0001
Baseline AHTKV-α	45.5 (13.1)	42.7 (13.8)	39.0 (11.7)	35.4 (9.0)	27.6 (8.8)	< 0.0001
Baseline-measured HtTKV slope	38.4 (13.5)	40.6 (13.6)	36.8 (11.5)	38.6 (14.9)	36.4 (11.1)	0.4773
Age at onset of disease manifestations (year)						
MIC based on AHTKV-α	49.8 (12.2)	41.9 (13.5)	40.4 (11.7)	31.4 (9.1)	25.4 (8.2)	< 0.0001
Baseline AHTKV-α	44.2 (13.0)	42.6 (12.6)	38.6 (12.1)	33.6 (9.8)	27.9 (8.7)	< 0.0001
Baseline-measured HtTKV slope	40.1 (13.7)	39.5 (12.7)	36.3 (12.8)	36.7 (12.4)	43.4 (11.5)	0.1260
Age at onset of HTN (year) (*n* = 194)						
MIC based on AHTKV-α	47.8 (11.8)	46.4 (11.5)	43.2 (10.7)	35.0 (7.8)	29.2 (6.8)	< 0.0001
Baseline AHTKV-α	48.2 (12.5)	44.7 (10.0)	42.3 (10.7)	36.4 (8.0)	31.9 (8.1)	< 0.0001
Baseline-measured HtTKV slope	44.4 (12.5)	41.4 (10.1)	40.4 (12.7)	38.6 (10.4)	36.6 (9.0)	0.0192
Hypertensive patients, % (*n* = 267)						
MIC based on AHTKV-α	55.6	64.6	86.5	77.3	95.5	0.0015
Baseline AHTKV-α	58.9	82.4	88.7	81.5	81.1	0.0021
Baseline-measured HtTKV slope	85.7	73.1	70.4	83.3	78.4	0.2445

Values are mean (SD) for continuous variables. Using MIC, subgroups A–E correspond to the original subgroups 1A–1E. Baseline age was age at the initial measurement of TKV. Family history and hypertension data were not available for 26 and 29 patients, respectively. *P* values were obtained by ANOVA for continuous variables and by Pearson Chi-squared test for categorical variables

*MIC* Mayo Clinic Image Classification for ADPKD, *AHTKV-α* age- and height-adjusted TKV growth rate as described in the text, *TKV* total kidney volume, *HtTKV* height-adjusted TKV, *eGFR* estimated glomerular filtration rate calculated using serum creatinine according to the Isotope Dilution Mass Spectrometry and Modification of Diet in Renal Disease (IDMS–MDRD) with the Japanese coefficient, *HTN* hypertension

### Correlations of eGFR slopes with biomarkers

**Fig. 4:**
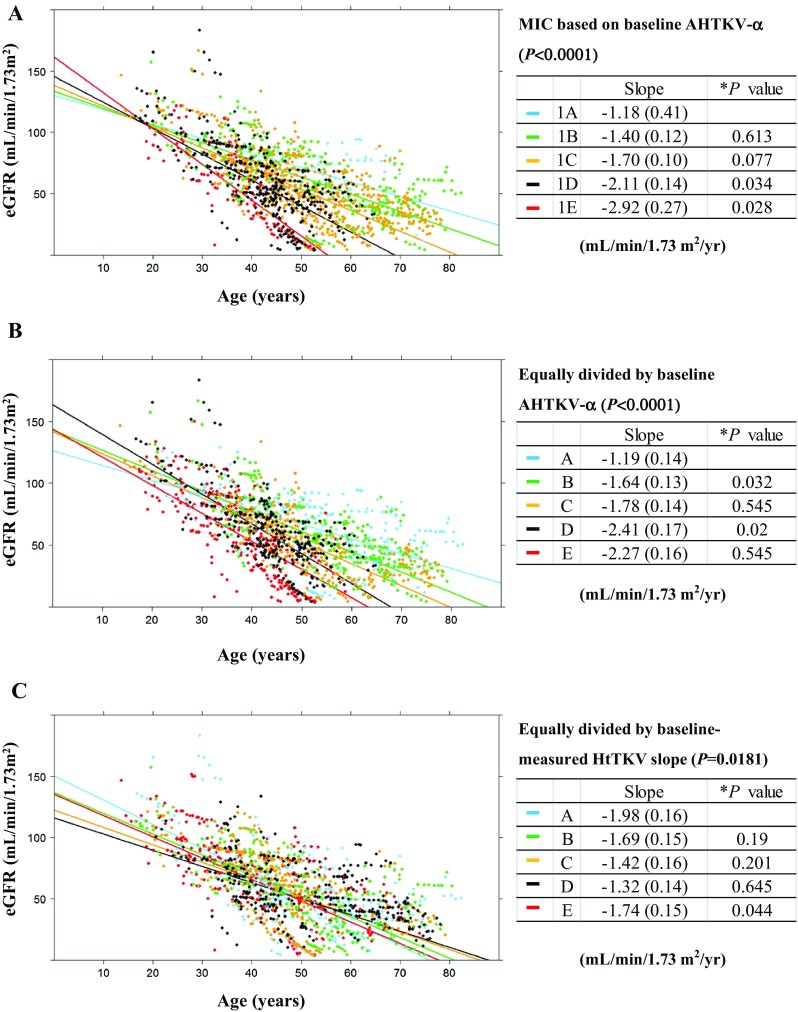
The eGFR slopes were evaluated longitudinally using a mixed-effects model for repeated measures, including age, sex, and interaction between group and age. Five subgroups were divided based on MIC definitions (**a**) and were divided equally by baseline AHTKV-α (**b**) and baseline-measured HtTKV slope (**c**). The limits of the divisions were the same as those in Fig. 3. *P values between adjacent subgroups were calculated using Benjamini and Hochberg’s multiple comparison procedure. For classifications in **a** and **b**, regression slopes were significantly different among the five subgroups (*P* < 0.0001) and generally increased from subgroups A–E. In contrast, inter-subgroup differences in eGFR slopes did not become steeper from subgroups A–E for classifications based on baseline-measured HtTKV slope (**c**)

From subgroups A–E, using MIC and AHTKV-α-based classifications, baseline age became younger and baseline eGFR became lower (all *P* < 0.0001) (Table 2). The eGFR slopes evaluated by a mixed-effects model became steeper from subgroups A–E using the AHTKV-α-based classification (*P* < 0.0001) (Table 2; Fig. 4a, b). Using classifications based on the baseline-measured HtTKV slope, the difference in eGFR slopes was significant; however, the slopes changed randomly from subgroups A–E (Table 2; Fig. 4c).

**Table 2:** eGFR-related data according to MIC and classifications equally divided by two baseline biomarkers

	A	B	C	D	E	*P* value
Patient number (male/female)						
MIC based on AHTKV-α (%/year)	11 (3/8)	69 (19/50)	107 (41/66)	78 (39/39)	24 (13/11)	0.1428
Baseline AHTKV-α (%/year)	57 (17/40)	58 (12/46)	58 (25/33)	58 (30/28)	58 (31/27)	0.0041
Baseline-measured HtTKV slope (%/year)	57 (15/42)	58 (24/34)	58 (24/34)	58 (24/34)	58 (28/30)	0.1815
Baseline age (year)						
MIC based on AHTKV-α	55.4 (3.8)	48.6 (1.5)	48.1 (1.2)	39.4 (1.4)	33.1 (2.5)	< 0.0001
Baseline AHTKV-α	49.8 (1.6)	50.6 (1.7)	48.4 (1.6)	41.7 (1.6)	34.5 (1.6)	< 0.0001
Baseline-measured HtTKV slope	47.4 (1.8)	46.1 (1.8)	43.9 (1.8)	46.5 (1.8)	41.5 (1.8)	0.1390
Baseline eGFR (mL/min/1.73 m^2^)						
MIC based on AHTKV-α	87.5 (6.2)	75.1 (2.5)	66.5 (2.0)	56.3 (2.4)	47.4 (4.3)	< 0.0001
Baseline AHTKV-α	78.4 (2.8)	72.4 (2.8)	65.7 (2.7)	59.1 (2.7)	50.2 (2.9)	< 0.0001
Baseline-measured HtTKV slope	65.8 (3.9)	65.8 (3.9)	66.9 (3.9)	62.2 (3.9)	68.8 (3.9)	0.8249
eGFR slope (mL/min/1.73 m^2^/year)						
MIC based on AHTKV-α	− 1.18 (0.41)	− 1.40 (0.12)	− 1.70 (0.10)	− 2.11 (0.14)	− 2.92 (0.27)	< 0.0001
Baseline AHTKV-α	− 1.19 (0.21)	− 1.64 (0.20)	− 1.78 (0.22)	− 2.41 (0.24)	− 2.27 (0.16)	< 0.0001
Baseline-measured HtTKV slope	− 1.98 (0.23)	− 1.69 (0.21)	− 1.42 (0.22)	− 1.32 (0.21)	− 1.74 (0.15)	0.0181

*P* value and mean (SE) were calculated using a least-square mean test for continuous variables and a likelihood ratio test for categorical variables. The slopes of eGFR were evaluated longitudinally using a mixed-effects model for repeated measures, including age, sex, and interaction between group and age. The slopes are adjusted for males. Baseline age was age at the initial measurement of eGFR

*MIC* Mayo Image Classification for ADPKD, *AHTKV-α* age- and height-adjusted TKV growth rate as described in the text, *TKV* total kidney volume, *HtTKV* height-adjusted TKV, *eGFR* estimated glomerular filtration rate calculated using serum creatinine according to the Isotope Dilution Mass Spectrometry and Modification of Diet in Renal Disease (IDMS–MDRD) with the Japanese coefficient

### Effects of sex and age on eGFR slopes

The eGFR slopes analyzed by a mixed-effects model were illustrated separately based on sex (Supplemental Fig. 3). Using AHTKV-α-based classifications (Supplemental Fig. 3a, b), the differences in the eGFR slopes were more evident among the five subgroups than between the two sexes.

**Fig. 5:**
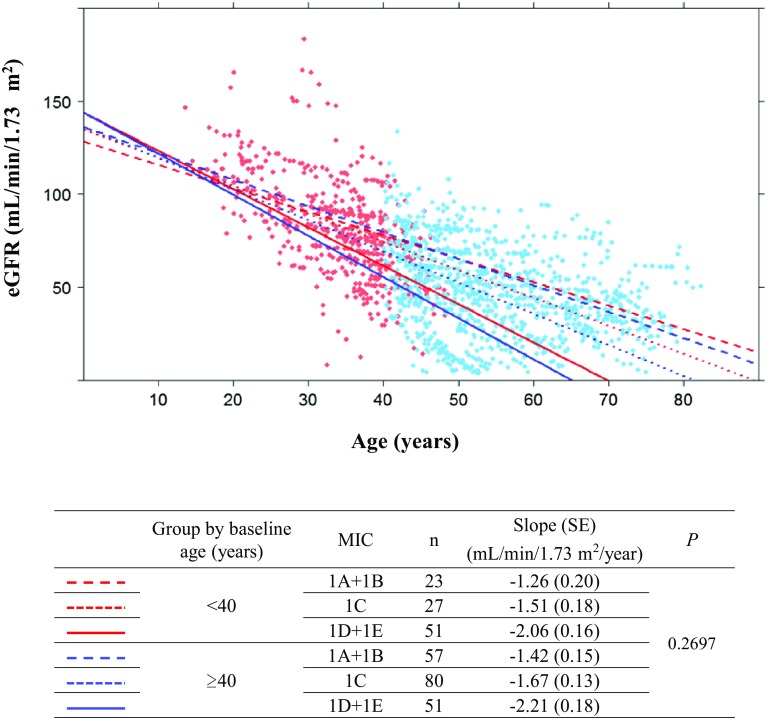
The eGFR slopes were evaluated using a mixed-effect model including sex, baseline age group (< 40, ≥ 40), MIC (1A + 1B, 1C, 1D + 1E), age, interaction between baseline age group and MIC, interaction between baseline age group and age, and interaction between MIC and age. The figures are adjusted for males. No significant age effects were observed on eGFR slopes

The effects of baseline age on eGFR slopes were analyzed using a mixed-effects model according to baseline ages and MIC subgroups (Fig. 5). Age subgroups were divided at 40 years (< 40 and ≥ 40), and five MIC subgroups were combined into three subgroups to avoid small groups. The eGFR slopes were not significantly different between the two age subgroups of the three MIC subgroups (*P* = 0.27).

### Renal survival analyses according to classifications based on two biomarkers

Renal survival was analyzed using the Cox multivariable proportional hazard model based on group and sex (Table 3). Because the total number of patients was small (*n* = 11), and no patient developed ESRD during the observation period in MIC subgroup 1A, the five subgroups were combined into three subgroups. Inter-subgroup hazard ratios were significantly different among subgroups 1A + 1B, 1C, and 1D + 1E. Patients were equally divided into three subgroups using three biomarkers for comparison purposes. In the AHTKV-α-based subgroups, renal survival hazard ratios were separated more clearly than in the baseline-measured HtTKV slope subgroups (Table 3). Cox proportional hazard model analyses comparing the sexes in relation to the subgroups showed no significant differences (Supplemental Table 2). Kaplan–Meier curves are illustrated in Supplemental Fig. 3. Curves were more clearly separated in the AHTKV-α-based subgroups (Supplemental Fig. 3a, b) than in the baseline-measured HtTKV slope subgroups (Supplemental Fig. 3c).

**Table 3:** Cox proportional hazard ratios according to MIC and classifications based on two biomarkers

Classification	Range	Unit	Subjects (*n*)	Cox proportional hazard analysis		
				Multivariate HR	95% CI	FDR-adjusted *P* value
MIC by AHTKV-α*						
1A + 1B	< 3.0	%/year	80	1		
1C	3.0–4.5		112	1.31	0.03–2.58	0.0443
1D + 1E	4.5 ≤		104	3.36	2.00–4.71	< 0.0001
Three equally divided subgroups for baseline AHTKV-α						
A	< 3.175	%/year	100	1		
B	3.175–4.53		98	1.62	0.46–2.79	0.006
C	4.53 ≤		98	3.49	2.26–4.73	< 0.0001
Three equally divided subgroups for baseline-measured HtTKV slope						
A	< 1.748	%/year	98	1		
B	1.748–7.91		99	0.11	− 0.71–0.92	0.799
C	7.91 ≤		99	− 0.62	− 1.66–0.41	0.361

Multivariate hazard ratios (HR) were for worse adjacent subgroups. *P* values were compared between adjacent subgroups

*MIC* Mayo Image Classification for ADPKD, *AHTKV-α* age- and height-adjusted TKV growth rate as described in the text, *TKV* total kidney volume, *HtTKV* height-adjusted TKV, *FDR* false discovery rate

### *PKD* genic and *PKD1* allelic influences on AHTKV-α

Baseline-measured HtTKV slope was not significantly different between *PKD1* and *PKD2* mutation carriers. However, baseline AHTKV-α was significantly higher for *PKD1* mutation carriers than *PKD2* mutation carriers (Table 4). In addition, baseline AHTKV-α was significantly higher for patients with truncating-type *PKD1* mutations than for patients with nontruncating-type *PKD1* mutations. *PKD* genic and *PKD1* allelic influences on HtTKV growth rates were documented using AHTKV-α.

**Table 4:** Influence of *PKD* mutations on baseline HtTKV-related biomarkers

Demographic data of 88 patients with pathogenic PKD mutations	All	*PKD1*	*PKD2*	*P* value
*N* (male/female)	88 (34/54)	77 (30/47)	11 (4/7)	0.8686
Age at baseline TKV measurement (year)	47.4 (13.6)	45.6 (12.6)	60.0 (14.3)	0.0008
TKV observation period (year)	4.9 (2.0)	4.76 (2.01)	6.07 (2.01)	0.0462
Baseline TKV (mL)	1851.7 (1042.7)	1901.3 (1056.0)	1503.9 (912.2)	0.2392
Baseline HtTKV (mL/m)	1135.6 (641.2)	1165.2 (651.1)	928.4 (548.9)	0.2542
Baseline eGFR (mL/min/1.73 m^2^)	60.9 (31.8)	61.6 (32.6)	56.1 (26.4)	0.5931

*PKD* genic influences on HtTKV growth biomarkers	All	*PKD1*	*PKD2*	*P value
Baseline AHTKV-α (%/year)	4.19 (1.31)	4.39 (1.26)	2.79 (0.78)	< 0.0001
Baseline-measured HtTKV slope (%/year)	4.55 (8.92)	4.61 (9.04)	4.16 (8.40)	0.9950

*PKD1* allelic influences on HtTKV growth biomarkers	All	Truncating-type	Nontruncating-type	*P value
*N* (male/female)	77	49 (22/27)	28 (8/20)	
Baseline AHTKV-α (%/year)	4.39 (1.25)	4.71 (1.14)	3.84 (1.27)	0.0300
Baseline-measured HtTKV slope (%/year)	4.61 (9.04)	5.49 (9.30)	3.06 (8.51)	0.5491

Values are means (SD) of variables. $$\begin{aligned} {\text{Measured HtTKV slope (}}\% /{\text{year)}} & =[{\text{difference in two consecutive HtTKV}}]/[{\text{HtTKV at an earlier point]}} \\ & \quad {\text{/[interval (year) of two measurements]}} \times {\text{100}}. \\ \end{aligned}$$

*TKV* total kidney volume, *HtTKV* height-adjusted TKV, *AHTKV-α* age- and height-adjusted TKV growth rate, *Truncating-type* truncating-type *PKD1* mutations, *Nontruncating-type* nontruncating-type *PKD1* mutations

*P values were calculated by multivariate analyses adjusting for group, sex and interaction between group and sex

## Discussion

Renal and extrarenal phenotypes of ADPKD are heterogeneous due to the varieties of *PKD* mutation types [13, 15, 16], modifying genetic factors [17], gender differences [18] and environmental factors [19]. Genetic, imaging, clinical, laboratory, and environmental predictors are factors that are related to determining ADPKD disease severity [20]. Genetic analysis is laborious and expensive [21] and genetic predictions of renal prognoses remain complicated and uncertain [22]. The combination of *PKD* mutation types and clinical scores (PROPKD score) was proposed for prognostic purposes [18]. This scoring system included clinical data that were age-dependently variable.

Imaging classifications were developed as biomarkers to predict renal prognoses for ADPKD. The CRISP study reported that six groups separated according to TKV (< 750, 750–1500 and ≥ 1500 mL) and age (< 30 and ≥ 30 years) were related to GFR slopes [4]. In the Tolvaptan Efficacy and Safety in Management of Autosomal Dominant Polycystic Kidney Disease and Its Outcomes (TEMPO) 3:4 trial, patients with a TKV of 750 mL or more were enrolled according to the CRISP results [11]. However, TKV is influenced by factors such as sex, body size, and age.

Extended observations of CRISP participants showed that HtTKV ≥ 600 mL/m predicted the risk of developing ESRD [6]. HtTKV reduced the influence of body size on TKV and improved prognostic accuracy. However, the age-dependent exponential increase in TKV [5] was not related with HtTKV.

Investigators from the Mayo Clinic Translational PKD Center proposed MIC for ADPKD to select optimal patients for enrollment in clinical trials [12]. MIC used a chart composed of log-converted HtTKV and age with four limits. Limits were defined based on estimated kidney growth rates of 1.5, 3.0, 4.5, and 6.0% per year. The MIC model depends on the assumption of exponential growth of the kidney volume at a rate of *α* (%/year) from an HtTKV_0_ of 150 mL/m at age 0. The MIC prediction model selected patients at high risk for rapid disease progression and improved clinical designs [23]. The present study confirmed that MIC subgroups were significantly correlated with the disease severity (Table 1), eGFR slope (Table 2; Fig. 4), and renal survival (Table 3 and Supplemental Fig. 3) in Japanese patients with ADPKD. The concept of age-dependent HtTKV growth was incorporated in the MIC prediction model and improved accuracy in predicting disease severity.

The exponential growth of TKV and prognostic ability of TKV for subjects older than 18 years were described by Grantham [5]. Figure 3 confirms the exponential pattern of HtTKV growth. The kidney weight-to-height ratio increases after birth and becomes stable at approximately 15–20 years in the normal population [24, 25]; therefore, the exponential increase in HtTKV after 15 years old is regarded to be due to renal cyst expansion [12].

During clinical trials, the percentage change in TKV or log-converted TKV was used as a primary or secondary endpoint [9–11]. As shown in Fig. 2 and Supplemental Fig. 1, the measured HtTKV slope and log-converted TKV slope fluctuated proportionally to baseline, whereas AHTKV-α stayed within a relatively narrow range. The actual change patterns of log-converted HtTKV for three patients who underwent 10 years of follow-up are shown in Supplementary Fig. 4. Because TKV increased in a wave-like pattern, the measured slopes fluctuated (from negative to positive), resulting in poor repeatability. In contrast, AHTKV-α remained within a relatively narrow positive range. No significant difference in TKV growth rate was recognized between *PKD1*- and *PKD2*-mutation carriers [15]. This result may be due to the poor repeatability of the measured TKV (or HtTKV) slope and log-converted TKV slope. The present study showed that the AHTKV-α was significantly higher for *PKD1* mutation carriers than for *PKD2* mutation carriers, and for truncating-type *PKD1* mutation carriers than for nontruncating-type *PKD1* mutation carriers (Table 4). Because AHTKV-α was an age-adjusted HtTKV growth rate, these results strongly suggest genic and allelic effects on the HtTKV growth rate in ADPKD.

The declining rate of GFR was hypothesized to increase after the end of compensation for the loss of the glomerular filtration function at approximately age 40 years [2]. The eGFR slope was compared between two age groups (< 40 and ≥ 40, Fig. 5). The analysis using the mixed-effect model did not show a significantly declining eGFR slope after age 40 in any MIC subgroup.

The declining eGFR slopes of five MIC subgroups overlapped where an eGFR of approximately 100–110 mL/min/1.73 m^2^ and an age of approximately 20 years crossed (Fig. 4a). The eGFR seems to start declining in a different manner from this common point of age and eGFR. The rate of the declining eGFR slope was significantly related to the HtTKV growth rate (AHTKV-α).

A limitation of this study was that it was retrospective. However, TKV was measured using the same volumetric method once every year beginning in 2007, and clinical data including eGFR and relevant clinical features were collected in a similar manner to that of a prospective cohort study at a single institute.

## Conclusions

AHTKV-α can be used as a sensitive marker of the HtTKV growth rate due to better repeatability than the measured HtTKV slope. MIC is a good renal prognostic marker applicable to Japanese patients also. PKD genic and allelic effects on the HtTKV growth rate were demonstrated using AHTKV-α but not using HtTKV slope. Further large-scale studies are required to validate the use of AHTKV-α as a sensitive marker in clinical trials.

## Electronic supplementary material

Below is the link to the electronic supplementary material.

Supplementary material 1 (DOCX 1036 KB)
