# Supplementary material for: Age- and height-adjusted total kidney volume growth rate in autosomal dominant polycystic kidney diseases
Source: Clin Exp Nephrol. 2018 Jul 26;23(1):100–11. doi: 10.1007/s10157-018-1617-8 (PMC6344392; doi:10.1007/s10157-018-1617-8)

# **Supplemental Tables**

## Supplemental Table 1. Brief summary of TKV and eGFR measurements

|  |  |  | Median (IQR) | Mean | SD |
| --- | --- | --- | --- | --- | --- |
| TKV measurements (1240 measurements of 296 patients) | | | |  |  |
|  | Observed period per patient | (year /patient) | 2.75 (1.08-5.25) | 3.30 | 2.37 |
|  | Frequency | (times/patient) | 4 (2-5) | 4.19 | 2.14 |
|  | Interval between measurements | (year) | 1.00 (0.83-1.08) | 1.06 | 0.66 |
| eGFR measurements (1495 measurements of 289 patients) | | | |  |  |
|  | Observed period per patient | (year/patient) | 2.58 (1.08-5.92) | 3.68 | 2.94 |
|  | Frequency | (time/patient) | 4 (3-7) | 5.29 | 3.20 |
|  | Interval between measurements | (year) | 0.92 (0.50-1.00) | 0.86 | 0.69 |

TKV, total kidney volume; eGFR, estimated glomerular filtration rate; IQR, interquartile range.

## Supplemental Table 2. Cox multivariate hazard ratios according to sex and classifications

| Classification | | Range | Male | | |  | Female | | | *P* for interaction |
| --- | --- | --- | --- | --- | --- | --- | --- | --- | --- | --- |
|  |  |  | Subjects (n) | HR (95% CI) | *P* value |  | Subjects (n) | HR (95% CI) | *P* value |  |
| MIC based on AHTKV-α* (%/year) | | | | | |  |  |  |  | 0.1840 |
|  | 1A + 1B | < 3.0 | 22 |  |  |  | 58 |  |  |  |
|  | 1C | 3.0 - 4.5 | 41 | 0.93 (0.09-20.08) | 0.96 |  | 71 | 5.41 (1.44-36.03) | 0.0107 |  |
|  | 1D + 1E | 4.5 ≤ | 49 | 7.85 (1.26-75.20) | 0.0273 |  | 55 | 7.64 (2.81-21.23) | <0.0001 |  |
| Equally divided 3 groups by AHTKV-α (%/year) | | | | | |  |  |  |  | 0.2320 |
|  | A | < 3.18 | 28 |  |  |  | 71 |  |  |  |
|  | B | 3.18 - 4.575 | 37 | 1.49 (0.14-32.37) | 0.74 |  | 61 | 6.67 (1.95-30.59) | 0.0021 |  |
|  | C | 4.575 ≤ | 47 | 7.41 (1.16-75.90) | 0.0337 |  | 52 | 6.26 (2.31-17.47) | 0.0004 |  |
| Equally divided 3 groups by baseline measured HtTKV slope (%/year) | | | | | |  |  |  |  | 0.6068 |
|  | A | < 1.7 | 28 |  |  |  | 71 |  |  |  |
|  | B | 1.7 -7.91 | 42 | 2.25 (0.28-45.73) | 0.46 |  | 56 | 1.11 (0.43-2.71) | 0.82 |  |
|  | C | 7.91 ≤ | 42 | 1.37(0.25-7.50) | 0.71 |  | 57 | 0.23 (0.03-0.90) | 0.0343 |  |

MIC, Mayo Image Classification for ADPKD; AHTKV-α, age- and height-adjusted TKV growth rate as described in the text; TKV, total kidney volume; HtTKV, height-adjusted TKV. Multivariate hazard ratios (HRs) were for adjacent worse subgroups. *P* for interaction was for difference in Cox hazard ratio between two genders.

# Supplemental Figures

## Supplemental Figure 1. Comparison of repeatability between AHTKV-α, measured HtTKV slope, and LogTKV slope.

The regression line of the LogTKV slope (baseline LogTKV slope vs. difference in LogTKV slope from baseline) was significant. R^2^=0.532, slope=-0.961 (SE, 0.035), and *P*<0.0001. The distribution range of the baseline LogTKV slope became narrower than the measured HtTKV slope due to log conversion, but significant bias remained.


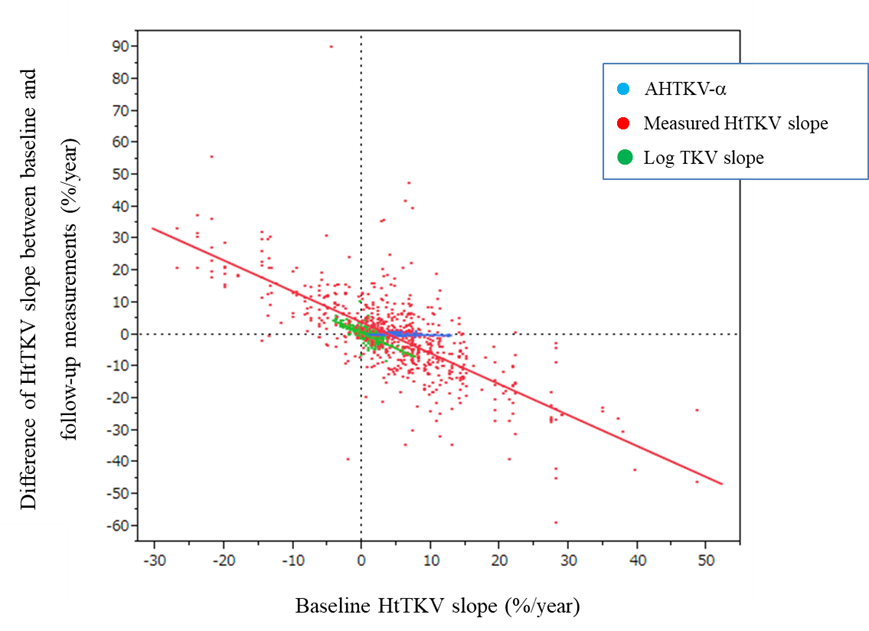


## Supplemental Figure 2. eGFR slopes according to classifications and sex.

eGFR slopes were evaluated using a mixed-effects model for repeated measures, including age and interaction between group and age. Slope is indicated by mean (SE).

A) MIC based on AHTKV-α


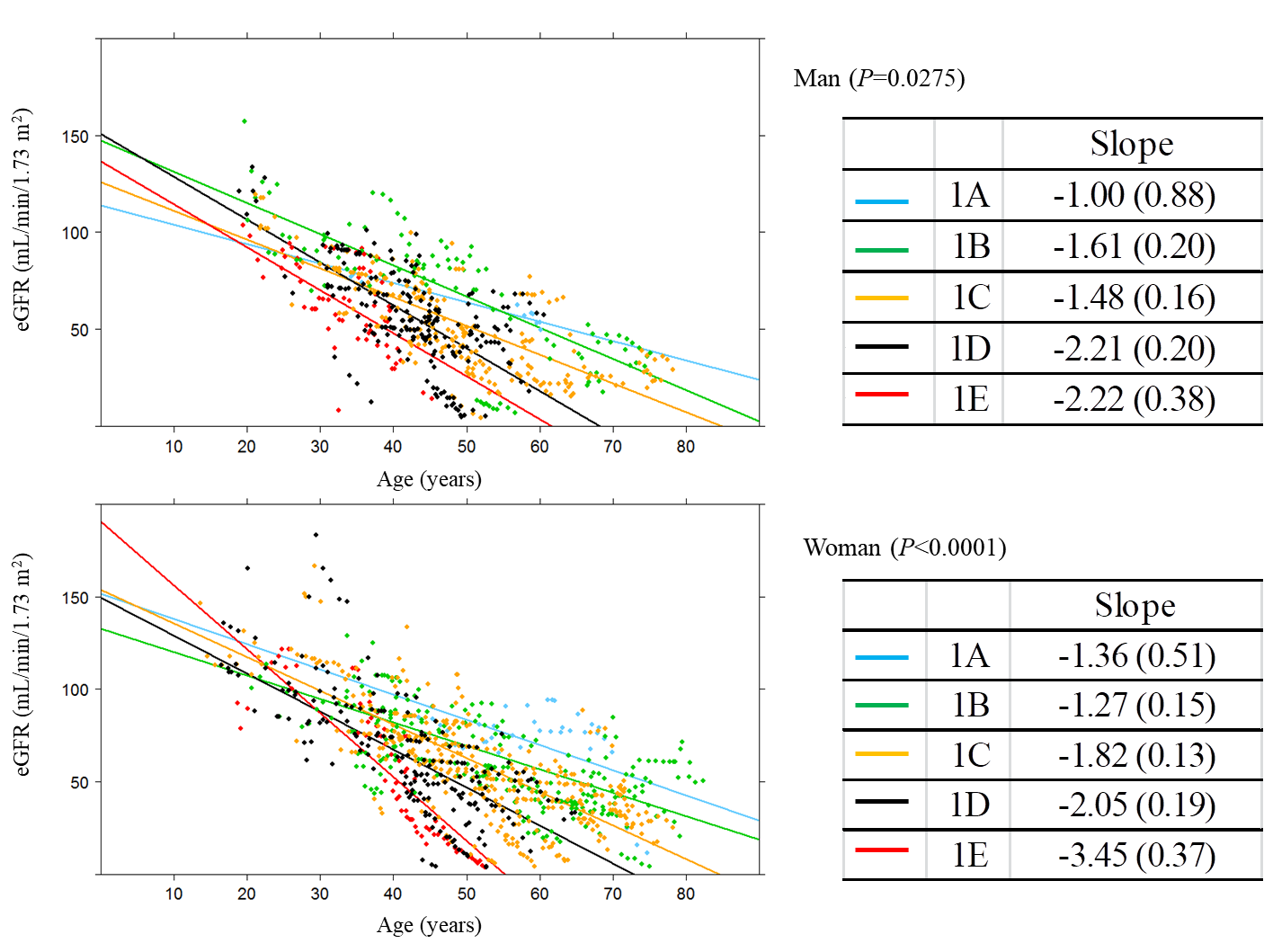


B) Five equally divided subgroups for AHTKV-α


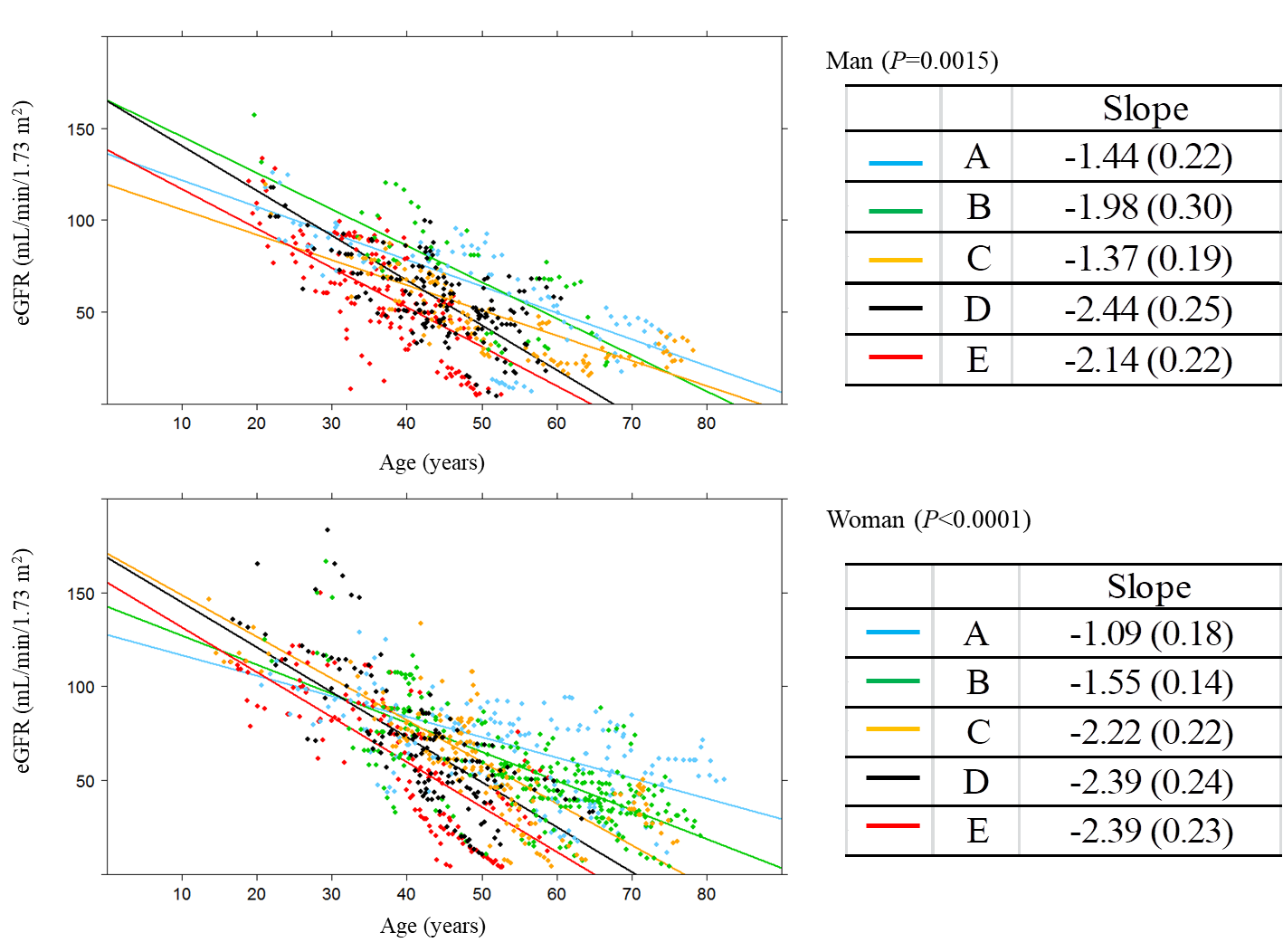


C) Five equally divided subgroups for baseline-measured HtTKV slope


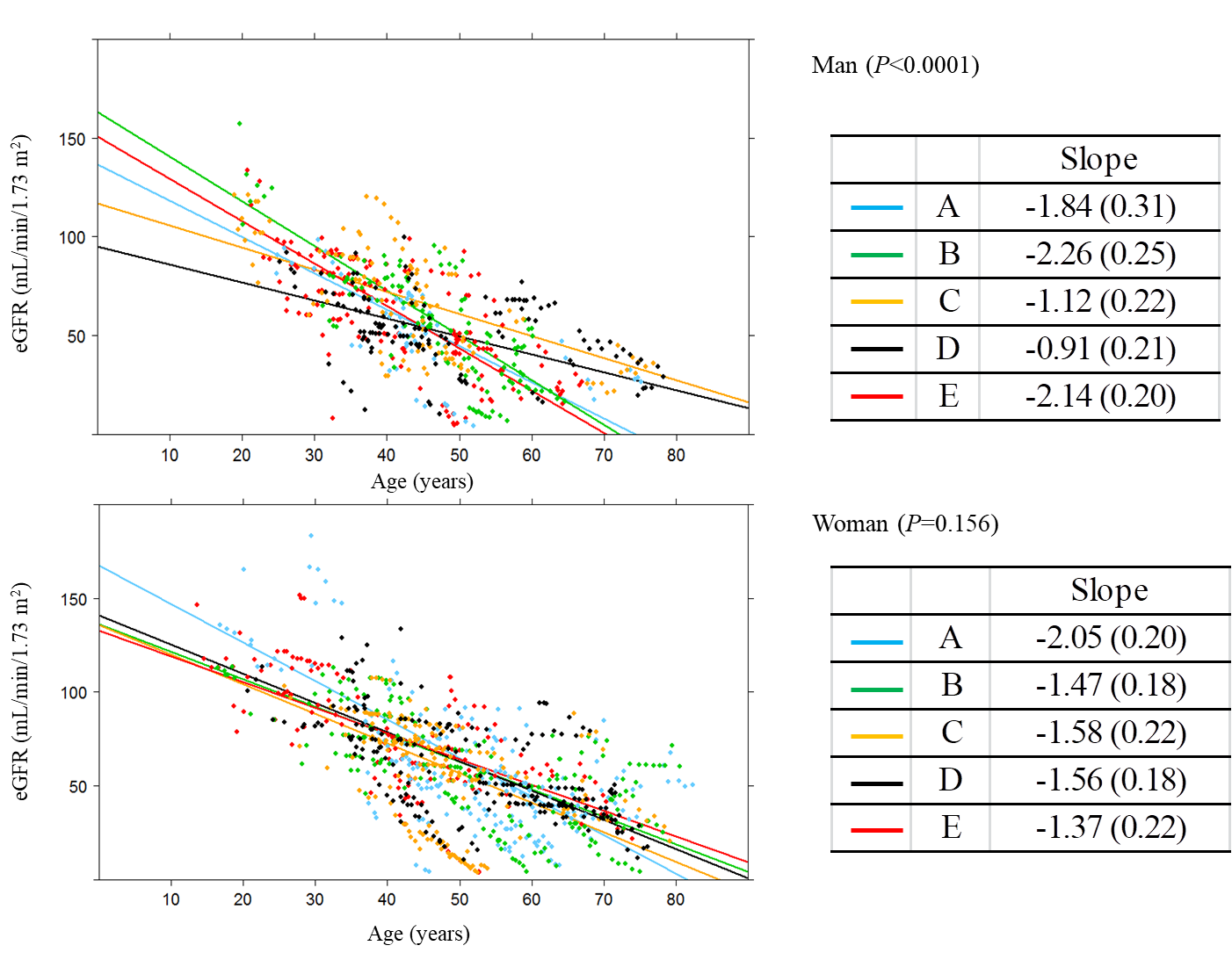


## Supplemental Figure 3. Kaplan-Meier renal survival curves until RRT.

The ranges of each subgroup are shown in Table 3.

A) Five MIC subgroups were combined into three subgroups (1A+1B, 1C, and D+1E)


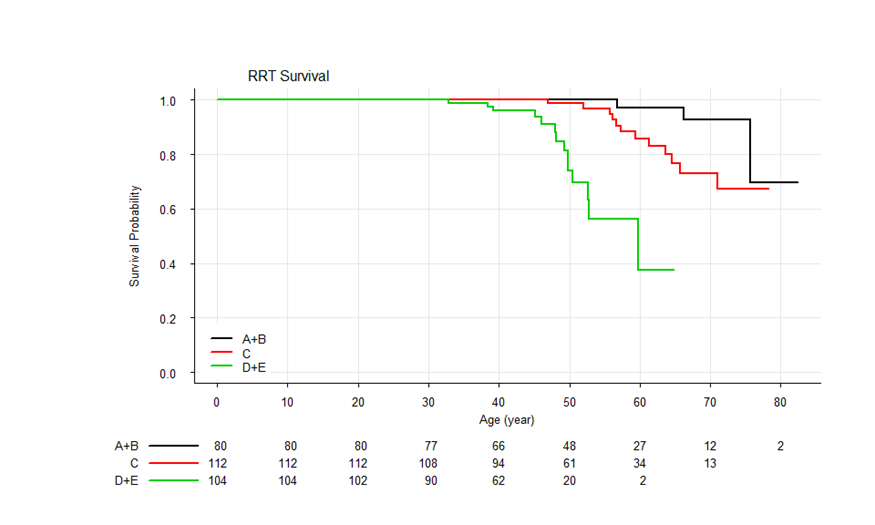


B) Three subgroups equally divided for AHTKV-α


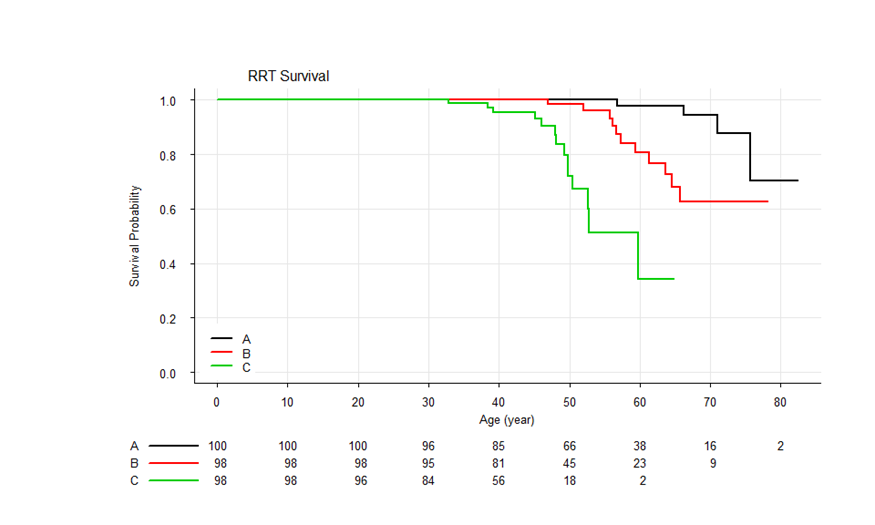


C) Three subgroups equally divided for the baseline-measured HtTKV slope


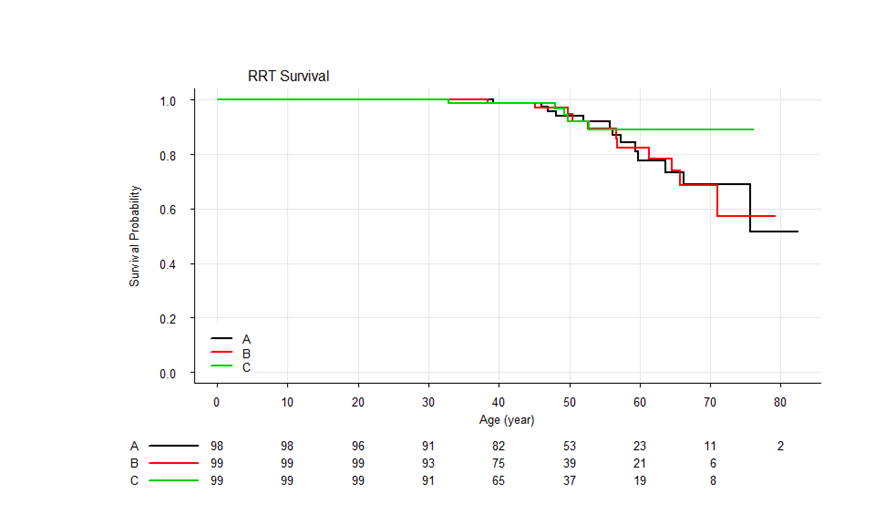


## Supplemental Figure 4. Actual pattern of HtTKV changes in three patients with TKV measured for 10 years.

Measured slopes fluctuated widely from positive to negative slopes. In contrast, AHTKV-α varied within a narrow range between 4.2% and 5.2 % per year, and changes remained within less than 1 % per year.


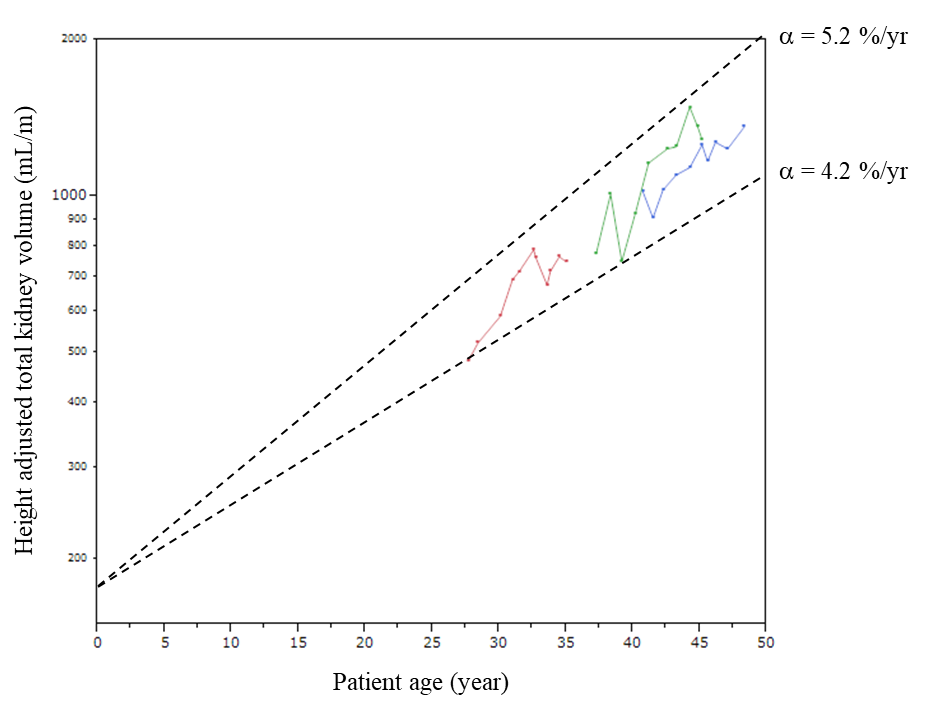

Supplement: Supplementary file 1 — Supplementary material 1 (DOCX 1036 KB) [file 10157_2018_1617_MOESM1_ESM.docx]
